# Understanding the role of coronary artery revascularization in patients with left ventricular dysfunction and multivessel disease

**DOI:** 10.1007/s10741-023-10335-0

**Published:** 2023-07-26

**Authors:** Vincenzo Acerbo, Arturo Cesaro, Gianmaria Scherillo, Giovanni Signore, Francesco Paolo Rotolo, Gianantonio De Michele, Francesco Scialla, Giuseppe Raucci, Domenico Panico, Fabio Fimiani, Elisabetta Moscarella, Felice Gragnano, Paolo Calabrò

**Affiliations:** 1 Department of Translational Medical Sciences, University of Campania “Luigi Vanvitelli”, 80131 Naples, Italy; 2 Division of Cardiology, A.O.R.N. “Sant’Anna e San Sebastiano”, Caserta, Italy

**Keywords:** Heart failure, PCI, CABG, HFrEF, CAD, Coronary revascularization

## Abstract

Coronary artery disease (CAD) is the most common cause of heart failure with reduced ejection fraction (HFrEF). Advances and innovations in medical therapy have been shown to play a crucial role in improving the prognosis of patients with CAD and HFrEF; however, mortality rate in these patients remains high, and the role of surgical and/or percutaneous revascularization strategy is still debated. The Surgical Treatment for Ischemic Heart Failure (STICH) trial and the Revascularization for Ischemic Ventricular Dysfunction (REVIVED) trial have attempted to provide an answer to this issue. Nevertheless, the results of these two trials have generated further uncertainties. Their findings do not provide a definitive answer about the ideal clinical phenotype for surgical or percutaneous coronary revascularization and dispute the historical dogma on myocardial viability and the theory of myocardial hibernation, raising new questions about the proper selection of patients who are candidates for coronary revascularization. The aim of this review is to provide an overview on the actual available evidence of coronary artery revascularization in patients with CAD and left ventricular dysfunction and to suggest new insights on the proper selection and management strategies in this high-risk clinical setting.

## Introduction

Coronary artery disease (CAD) is the most common cause of heart failure with reduced ejection fraction (HFrEF) globally [1] and the leading cause of global mortality [2]. Therapeutic management of patients with CAD represents a fascinating challenge of our time. Rapid progress in guideline-directed optimal medical therapies (OMT), including pharmacological and implantable device therapies, has drastically improved clinical outcomes for patients with ischemic cardiomyopathy [2–5]. However, beyond medical therapy alone, there is still heated debate around the prognostic impact of coronary artery revascularization in this clinical setting. The Surgical Treatment for Ischemic Heart Failure (STICH) trial [6] and the Revascularization for Ischemic Ventricular Dysfunction (REVIVED) trial [7] investigated, in a randomized controlled fashion, the role of coronary revascularization via coronary artery bypass graft (CABG) or percutaneous coronary intervention (PCI), respectively, among patients with left ventricular (LV) dysfunction. Their results question the historical dogma on myocardial viability and the theory of myocardial hibernation; rather than clarify, these findings create uncertainty regarding the ideal patient: individuals who would benefit the most from surgical or percutaneous coronary revascularization. In this review, we discuss the role of coronary revascularization in ischemic cardiomyopathy and provide practical suggestions for the selection and management of patients with this condition in clinical practice.

## The STICH trial

The STICH trial is a randomized controlled clinical trial that enrolled 1212 patients with left ventricle ejection fraction (LVEF) of ≤ 35% and CAD amenable to CABG to test the hypothesis that CABG combined with OMT can improve survival more than OMT alone [6]. The patients were randomly assigned to receive CABG and OMT (a CABG group of 610 patients) or OMT alone (a OMT group of 602 patients). Patients were ineligible for inclusion in the trial if they had a left main coronary artery stenosis of ≥ 50% or a Canadian Cardiovascular Society class III or IV angina. Over a median follow-up period of 56 months, no significant difference was observed in the primary endpoint of all-cause mortality between the CABG group (36%) and the OMT group (41%), despite a trend toward better clinical outcomes with CABG combined with OMT than with OMT alone (hazard ratio [HR] = 0.86, 95% confidence interval [CI] = 0.72–1.04, *P* = 0.12). Notably, the CABG group had a lower rate of death from cardiovascular causes than the OMT group (28% vs. 33%; HR, 0.81; 95% CI, 0.66 to 1.00; *P* = 0.05) [6] and an improved composite endpoint of death from any cause and composite endpoint of hospitalization for heart failure compared with the OMT group (58% vs. 68%) (HR = 0.74, 95% CI = 0.64–0.85, *P* < 0.001) [6], which were prespecified secondary endpoints [8].

### The STICH extension study (STICHES)

The STICHES trial [9] is a prespecified extension of the STICH trial [6] with an extended follow-up of an additional 5 years. In contrast with the first 5-year report [6], the rate of death from any cause was significantly lower in the CABG group (58.9%) than in the OMT group (66.1%) based on the data from the STICHES trial, which spanned a median follow-up period of 9.8 years (HR = 0.84, 95% CI = 0.73–0.97, *P* = 0.02) [9]. Furthermore, the secondary outcome analyses show consistent benefits from CABG: death from cardiovascular causes occurred in 246 patients (40.5%) in the CABG group and 297 patients (49.3%) in the OMT group (HR = 0.79, 95% CI = 0.66–0.93, *P* = 0.006), and death from any cause or hospitalization for cardiovascular causes occurred in 467 patients (76.6%) in the CABG group and 524 patients (87.0%) in the OMT group (HR = 0.72, 95% CI = 0.64–0.82, *P* < 0.001) (Table 1) [9].

**Table 1:** Trial details, clinical characteristics, and medical therapies of the patients at baseline^a^

**Trial details**	**STICHES trial**		**REVIVED-BCIS2 trial**	
Enrolled patients	1212		700	
Intermediate median follow-up in years (IQR)	4.6 (4–5.6)		3.4 (2.3–5)	
Extended median follow-up in years (IQR)	9.8 (9.1–11)			
**Clinical characteristics and medical therapies at baseline**	**CABG group (*N* = 610)**	**OMT group (*N* = 602)**	**PCI group (*N* = 347)**	**OMT group (*N* = 353)**
Median age (IQR)—yr	60 (54–68)	59 (53–67)	70 (61–79)	69 (60–78)
Male sex—no. (%)	537 (88)	527 (88)	302 (87)	312 (88)
Medical therapies at baseline—no. (%)				
Angiotensin receptor neprilysin inhibitor	0 (0)	0 (0)	15 (16)	23 (26)
Angiotensin converting enzyme inhibitor	514 (84)	482 (80)	237 (69)	235 (67)
Angiotensin receptor blocker	53 (9)	62 (10)	56 (16)	59 (17)
Mineralocorticoid receptor antagonist	280 (46)	276 (46)	176 (51)	170 (48)
Beta-blocker	507 (83)	529 (88)	315 (91)	319 (90)
Implantable cardioverter defibrillator	15 (2)	14 (2)	47 (55)	35 (45)
CCS angina class—no. (%)^b^				
No angina	217 (36)	225 (37)	228 (66)	236 (67)
I or II	361 (59)	351 (58)	111 (32)	107 (30)
III or IV	32 (5)	26 (5)	7 (2)	8 (2)
Left ventricular ejection fraction—%	27 (22–33)	28 (22–34)	27 (20–34)	27 (20–34)
Coronary artery disease (CAD) characteristic				
Left main CAD—no. (%)	18 (3)	14 (2)	50 (14)	45 (13)
Three-vessel CAD—no. (%)	228 (37)	214 (36)	133 (38)	148 (42)
Two-vessel CAD—no. (%)	233 (38)	229 (38)	178 (51)	166 (47)

^a^*CABG* coronary artery bypass grafting, *PCI* percutaneous coronary intervention, *IQR* interquartile range

^b^The Canadian Cardiovascular Society (CCS) angina classes range from I to IV, with higher classes indicating more disabling pain due to angina

#### Impact of age on long-term prognosis after CABG

Combining CABG with OMT extended median survival by 1.5 years with a 16% relative risk reduction and an 8% absolute risk reduction in all-cause mortality [9]. Notably, the impact of CABG on clinical outcomes may be influenced by age at the time of surgery. In a post hoc analysis of the STICHES study, Petrie et al. [10] categorized the STICHES cohort according to age-based quartiles (Q1 ≤ 54 years, Q2 > 54 and ≤ 60 years, Q3 > 60 and ≤ 67 years, and Q4 > 67 years) to investigate whether patients derived the same benefits from CABG irrespective of age [11]. In this subanalysis, using CABG combined with OMT offered a significant reduction in cardiovascular mortality across all age quartiles, indicating that younger patients experienced a greater benefit than older patients. In addition, the study results do not indicate any all-cause mortality benefits in patients > 67 years of age (HR = 0.82, 95% CI = 0.63–1.06). These observations can be attributed to several contributing factors, e.g., mortality among older patients was more likely due to non-cardiovascular causes. Indeed, cardiovascular-related deaths comprised a relatively small proportion of deaths (62%) in the quartile with the oldest patients, compared with 79% in the quartile with the youngest patients [10]. In patients who are advanced in age, CABG may not reduce (and may even increase) incidences of non-cardiovascular deaths due to the significant burden of comorbidities, which elevates the risk of postoperative complications and non-cardiovascular fatalities [10]. These data suggest that a selective (age-based) approach may be beneficial, as most CABG-related benefits are observed in patients < 60 years old with three-vessel CAD and severely impaired LV function [12]. Based on the data, a potential limitation of the STICH trial is the young age of the participants (median age: 60 years), which contrasts with contemporary clinical practice in which the age of patients receiving a heart failure diagnosis often exceeds 75 years [13]. Thus, it remains unclear whether the CABG risk–benefit ratio is favorable among patients aged > 60 years, who were underrepresented in the STICH trial.

### The theory of myocardial hibernation

Myocardial viability testing is crucial in patients with ischemic cardiomyopathy because, based on the historical model of myocardial hibernation, it facilitates the identification of patients with viable myocardium—who are more likely to benefit from coronary revascularization. Myocardial hibernation is a dynamic process characterized by reversible contractile dysfunction triggered by recurrent ischemia, and it describes an adaptive downregulation of myocardial function that favors myocyte survival [14]. This phenomenon involves different stages of ischemic dysfunction, from myocardial stunning (i.e., hours or days of contractile dysfunction following a short ischemic period) to myocardial hibernation (i.e., prolonged impairment of contractility due to recurrent episodes of nonlethal ischemia, which can be partially or completely restored to normal if the myocardial oxygen supply–demand relationship is favorably altered) [12] to scar formation (advanced hibernating myocardium is replaced with fibrosis as a continuum process) [15, 16].

#### Myocardial viability substudy

In patients with ischemic cardiomyopathy and multivessel CAD, myocardial viability has traditionally been considered a key factor in making the decision to perform revascularization [17–19]. In the STICH trial, a prespecified analysis was used to test the hypothesis that viability testing is a powerful tool for predicting improvements in LV function and survival after undergoing CABG combined with OMT compared with OMT alone [8]. The viability substudy included 601 patients who were prospectively assessed for myocardial viability using single-photon emission computed tomography (SPECT), dobutamine stress echocardiography (DSE), or both. The patients were divided into two groups: predominantly viable myocardium and predominantly nonviable myocardium [8]. Surprisingly, no significant association was observed between the evidence or absence of evidence of myocardial viability and the beneficial effects of CABG combined with OMT over OMT alone (*P* = 0.34 for interaction) [20]. In addition, predominantly viable myocardium was associated with an improvement in LV function irrespective of treatment (CABG vs. OMT), but this improvement did not translate to a long-term survival benefit [20].

#### Resizing the prognostic potential of contractile recovery

Based on additional findings from the myocardial viability substudy in the STICH trial [20], LV functional recovery should no longer be considered the most critical mechanism for improving clinical outcomes following CABG, as prevention of further myocardial injury, protection of the residual viable myocardium from future acute coronary events, and prevention of sudden cardiac deaths due to fatal ventricular arrhythmias probably contribute significantly to improving clinical outcomes [20]. Revascularization ensures the functional and electrical stability of myocytes, and this is achieved independently of LV systolic improvement. These data suggest that ensuring the blood flow into the hibernated myocardium distal to chronic coronary occlusions and the subsequent prevention of further acute ischemic and arrhythmic events is the main benefit of coronary artery revascularization in patients with ischemic cardiomyopathy [24].

### Inducible myocardial ischemia substudy

Due to the lack of data on the prognostic impact of myocardial ischemia in patients with CAD and LV dysfunction, the second substudy of the STICH trial [25] was conducted to test the hypothesis that the presence of inducible myocardial ischemia can identify those patients who can derive the greatest benefit from CABG over OMT alone. This prespecified analysis included 399 patients who were prospectively assessed for inducible myocardial ischemia using SPECT or DSE. Ischemia was analyzed both by dichotomous fashion and as a continuous variable: the stress testing identified 256 patients (64% of the study population) with inducible myocardial ischemia and 143 patients (36% of the study population) without demonstrable myocardial ischemia; conversely when ischemia was analyzed as a continuous variable, the percent ischemic myocardium was 12.2 ± 11.5% for the STICH patients enrolled, 199 of whom showed moderate-to-severe myocardial perfusion abnormalities (i.e., involving > 10% of the LV) [25]. However, similarly to the myocardial viability substudy, no association was observed between inducible myocardial ischemia and the patients’ adverse outcome for all-cause mortality (*P* 0.28), cardiovascular mortality (*P* 0.07), or death plus cardiovascular hospitalization (*P* 0.79). Furthermore, no interaction was found between the treatment effects of CABG over OMT and the presence or absence of myocardial ischemia for all-cause mortality or either of the secondary endpoints [25]. These results, as suggested in a non-prespecified post hoc analysis of the same study [26], failed to demonstrate any favorable effect of ischemia-guided myocardial revascularization in patients with ischemic cardiomyopathy. Therefore, despite the European guidelines recommendations [27], those findings suggest that patients with ischemic cardiomyopathy should be referred for CABG based on clinical criteria other than the presence of myocardial ischemia [26].

## Positive suggestions about ischemia-guided myocardial revascularization

In partial contradiction to previous evidence from the nuclear substudy of the STICH trial, a subanalysis of the ISCHEMIA (International Study of Comparative Health Effectiveness With Medical and Invasive Approaches) trial [28] suggested that ischemia-guided myocardial revascularization may have a beneficial effect in patients with a history of HF or left ventricular dysfunction (LVD). This substudy not only revealed that patients with stable ischemic heart disease and HF/LVD had a worse prognosis than patients without HF/LVD, but also that patients with HF/LVD who underwent an initial revascularization strategy had better clinical outcomes than those with an initial pharmacological strategy, whereas no difference was present in patients without HF/LVD. Nevertheless, it should be noted that the favorable outcomes of initial invasive treatment strategy identified in the ISCHEMIA trial [28] were limited to a subgroup of only 28 patients with HF and mildly-to-moderately decreased left ventricular ejection fraction (35 to 45%), which renders the findings at best, hypothetical [29]. It must also be underlined that this evidence, although only a suggestion, was obtained despite the fact that patients enrolled in the ISCHEMIA trial were treated with better medical therapy than STICH patients.

Further evidence suggesting the clinical relevance of ischemia testing in guiding clinical decisions is provided by an interesting retrospective observational analysis [30] which indicates a potential advantage of ischemia-guided revascularization in the presence of a severe ischemic burden (> 15% of the LV myocardium) in patients with stable CAD without LVEF selection.

To date, no randomized controlled trial has been conducted to assess the prognostic significance of myocardial ischemia in patients with ischemic cardiomyopathy; therefore, based on the current evidence, there is no compelling justification to perform myocardial revascularization in patients with ischemic cardiomyopathy and suitable coronary anatomy solely on the basis of the presence and severity of inducible myocardial ischemia.

## Modern application of viability and ischemic testing in ischemic cardiomyopathy

Based on the myocardial viability substudy and the inducible myocardial ischemia substudy in the STICH trial, the role of viable or ischemic myocardium for treatment selection and prognostication in patients with ischemic cardiomyopathy appears uncertain because no relationship was detected between the benefits of CABG and the results of the stress testing. To further emphasize the nonspecificity of the information provided by the viability testing, it should be noted that evidence of extensive viability has been shown to predict patient response not only to revascularization but also to medical therapy (both pharmacological [21] and cardiac resynchronization therapy [22]). Furthermore, the viability substudy findings highlight that changes in LV function are not a valid surrogate endpoint for long-term survival. On the other hands, surgical myocardial revascularization should be beneficial only in the presence of severe inducible myocardial ischemia (> 15% of the LV); conversely, in the remaining clinical cases, ischemia-guided coronary revascularization appears to offer no prognostic benefit but only an improvement in clinical symptoms refractory to OMT.

Based on the available evidence, Panza et al. [23] and Liga et al. [29] recently investigated the contemporary application of viability and ischemic testing in this clinical setting and recommended a paradigm shift in the management of patients with ischemic cardiomyopathy who are eligible for surgical revascularization [20, 23]. A vital concept in their proposal is that the amount of viable or ischemic myocardium should be assessed as a continuous variable that is unsuitable for dichotomous classification; therefore, patients should no longer be dichotomized roughly into those “with viability or inducible-ischemia” versus those “without viability or inducible-ischemia.” To make viability testing particularly useful for treatment and prognosis, the decision-making process regarding CABG feasibility should not focus on a prespecified myocardial viability threshold but on (i) the amount and distribution of viable segments and (ii) the concordance between viable segments and vessels that are suitable for surgical revascularization. In addition, an inducible myocardial ischemia testing should only be justified in the case of anginal symptoms or equivalents and surgical revascularization should only be pursued in the case of severe inducible myocardial ischemia. Similarly, the patient could be a candidate for percutaneous revascularization with the understanding that such a procedure would not improve prognosis but only quality of life. This approach may improve the selection of ideal candidates for whom CABG can effectively reduce the risk of fatal myocardial infarction and life-threatening ventricular arrhythmias and, ultimately, improve survival.

## The REVIVED-BCIS2 trial

The REVIVED British Cardiovascular Intervention Society (BCIS) 2 (REVIVED-BCIS2) trial [7] was a randomized controlled trial that, based on the evidence garnered from the STICH trial, tested the intriguing hypothesis that PCI combined with OMT can provide a greater survival benefit than OMT alone in patients with ischemic cardiomyopathy, thus overcoming procedural and early CABG-related hazards. The study enrolled 700 patients with LVEF ≤ 35%, extensive CAD (indicated by a BCIS jeopardy score of ≥ 6), and evidence of myocardial viability in ≥ 4 dysfunctional segments amenable to PCI. The patients were randomly assigned to either undergo PCI along with medical therapy (a PCI group of 347 patients) or to receive medical therapy alone (an OMT group of 353 patients). Patients were ineligible for participation if they had experienced an acute myocardial infarction during the 4 weeks before the random group assignment or if they had experienced acute decompensated heart failure or ventricular arrhythmias within 72 h before the random group assignment. Over a median follow-up period of 41 months (similar to that in the STICH trial), no statistically significant difference was observed in hospitalization for heart failure or the primary composite outcome of death from any cause between the PCI group (37.2%) and the OMT group (38%) (HR = 0.99, 95% CI = 0.78–1.27, *P* = 0.96) (Table 1). Major secondary endpoints included LVEF at 6 and 12 months, for which there were similar changes in the two groups at 6 months (mean difference = − 1.6 percentage points, 95% CI = − 3.7 to 0.5) and at 12 months (mean difference = 0.9 percentage points, 95% CI = − 1.7 to 3.4). Quality-of-life scores were higher for the PCI group at 6 and 12 months, but this difference diminished at 24 months.

## Analogies and differences between populations

Although the REVIVED [7] and STICH [6] trials were both designed to evaluate the benefits of revascularization in patients with ischemic cardiomyopathy, the patient populations in these studies differed for several reasons (Table 2). Compared with the STICH cohort, the REVIVED patients were 10 years older on average (mean age of 70 years); had a greater CAD burden (including left main CAD); received more contemporary guideline-directed medical therapy, including recent disease-modifying drugs (i.e., mineralocorticoid receptor antagonists [MRAs] and angiotensin receptor or neprilysin inhibitors); and were more frequently treated using device therapies (i.e., cardiac resynchronization therapy or implantable intracardiac defibrillators [ICDs]). The STICH study population was recruited between 2002 and 2007, and at that time, the only recommended class I medical therapies for HFrEF were angiotensin-converting enzyme inhibitors, beta-blockers, and digitalis [31]. Furthermore, the benefits of ICDs for primary prevention were initially reported in 2004–2005. High baseline risk features combined with improved medical therapy may at least partly explain the similar clinical outcomes of the REVIVED-BCIS2 trial and the STICH trial. Therefore, a contemporary randomized trial comparing CABG vs. PCI vs. OMT is probably needed to reconcile the different estimated therapeutic effects of surgical revascularization and percutaneous revascularization in STICH(ES) and the REVIVED-BCIS2, respectively, in light of these two studies employing different study populations and co-interventions [6, 7].

**Table 2:** Comparison of outcomes in the STICHES trial and REVIVED-BCIS2 trial

**Outcome**	**STICHES trial**				**REVIVED-BCIS2 trial**			
	**CABG group (*N* = 610)**	**OMT group (*N* = 602)**	**CABG effect (95% CI)**^**a**^	***P* value**	**PCI group (*N* = 347)**	**OMT group (*N* = 353)**	**PCI effect (95% CI)**^**a**^	***P* value**
Death from any cause at intermediate follow-up—no. (%)	218 (36)	244 (41)	0.86 (0.72–1.04)	0.12	110 (31.7)	115 (32.6)	0.98 (0.75–1.27)	
Death from any cause or hospitalization for heart failure at intermediate follow-up—no (%)	290 (48)	324 (54)	0.84 (0.71–0.98)	0.03	129 (37.2)	134 (38)	0.99 (0.78–1.27)	0.96
Death from cardiovascular causes at intermediate follow-up—no (%)	168 (28)	201 (33)	0.81 (0.66–1.00)	0.05	76 (21.9)	88 (24.9)	0.88 (0.65–1.20)	
Death from any cause at extended follow-up—no. (%)	359 (58.9)	398 (66.1)	0.84 (0.73–0.97)	0.02				
Death from any cause or hospitalization for heart failure at extended follow-up—no (%)	404 (66.2)	450 (74.8)	0.81 (0.71–0.93)	0.002				
Death from cardiovascular causes at extended follow-up—no (%)	247 (40.5)	297 (49.3)	0.79 (0.66–0.93)	0.006				

^a^CABG and PCI effects are hazard ratios

## An in silico model to emulate a clinical trial comparing CABG or PCI for heart failure

In order to bridge the knowledge gap and to provide information on feasibility of a randomized controlled trial, Pathak et al. [32], using an in silico model, attempted an intriguing comparison between surgical and percutaneous revascularization in patients with multivessel CAD and heart failure (HF) to assess their effectiveness in reducing 5-year all-cause mortality or cardiovascular hospitalization. After matching the target population of 13,519 HF patients, identified form hospital episode statistics (HES) database in England, with individual patient data from the STICH trial, the authors, using recent highly computational statistical methods, created two non-randomized intervention groups from the emulated trial cohort, a CABG group of 1174 patients and a complex PCI group of 872 patients: their results suggest a significantly lower rates of death and cardiovascular hospitalization at 5 years among HF patients undergoing CABG when compared with complex PCI (51.1% in the CABG group and 70.0% in the PCI group) [32]. Despite the in silico analysis limitations, these data not only underline that a superiority randomized controlled trial of CABG vs. PCI is feasible but also support the CABG as the treatment of choice in patients with ischemic cardiomyopathy eligible for surgery [33]. However, even if recent findings support CABG strategy rather than PCI in HFrEF patients, European guidelines [2, 27] show discordant indications. Hence, despite European guidelines on myocardial revascularization [27] give a strong recommendation for CABG with a moderate level of evidence (class I, level B), heart failure guidelines [2] provide a rather weak recommendation with the lowest level of evidence for CABG (class IIb, level C).

## Current evidence and major gaps in knowledge

Although longer-term follow-up data from the REVIVED trial are awaited to provide more solid evidence, the REVIVED-BCIS2 trial, in partial disagreement with the results derived from the subanalysis of the ISCHEMIA trial [34–37] for patients with HF and mildly reduced ejection fraction (HFmrEF), proved that a percutaneous coronary revascularization strategy should not be superior to a more-conservative approach for improving survival in asymptomatic or paucisymptomatic patients with chronic CAD and LVEF ≤ 35% and evidence of myocardial viability.

Although the overall incidence of myocardial infarction (MI) was similar in the two groups in the REVIVED-BCIS2 trial, almost twice as many spontaneous MIs were experienced in the OMT group as in the PCI group (9% vs. 5%), and this observation is potentially relevant because spontaneous MIs may have a worse impact on cardiovascular outcome than periprocedural MIs (experienced by ∼4% of the PCI group) [38, 39]. In addition, compared with the OMT group, there were fewer instances of appropriate ICD therapy in the PCI group (14.0% vs. 5.9% at 24 months, HR = 0.42, 95% CI = 0.17–1.06), indicating a potential beneficial effect of a reduction in the residual ischemic burden on the risk of arrhythmia [38].

However, several open questions remain. First, LV dysfunction and CAD are two different clinical conditions that can coexist without necessarily being related, especially in cases of severe ventricular dysfunction in the absence of extensive CAD (i.e., a two-vessel or single-vessel disease)—which was the situation for about half the REVIVED trial patients [40]. A severely depressed EF may also be secondary to valvular heart disease or myocarditis, or may simply be due to idiopathic cardiomyopathy. Therefore, the crucial question that every clinician should ask himself or herself and subsequently verify via correlating noninvasive and invasive physiological testing is whether the observed CAD is merely coincidental or the primary cause of ventricular dysfunction. Second, there is no information available on the anatomical and functional significance of coronary artery stenosis or its association with ischemic or viability testing [38]. Additional analyses and follow-up data from the REVIVED-BCIS2 trial are much awaited—especially regarding myocardial viability and LV functional recovery—to draw more solid conclusions and assess implications for clinical practice. Finally, it is necessary to specify that the limited data available from these RCTs on the efficacy of myocardial revascularization in patients with ischemic cardiomyopathy (ICM) hinder any conclusive determination of its benefits. Even the most reliable available evidence from non-randomized subanalyses of studies such as STICH are inadequate to draw definitive conclusions on the use of viability imaging for the management of patients with MCI [29].

## Conclusions

**Fig. 1:**
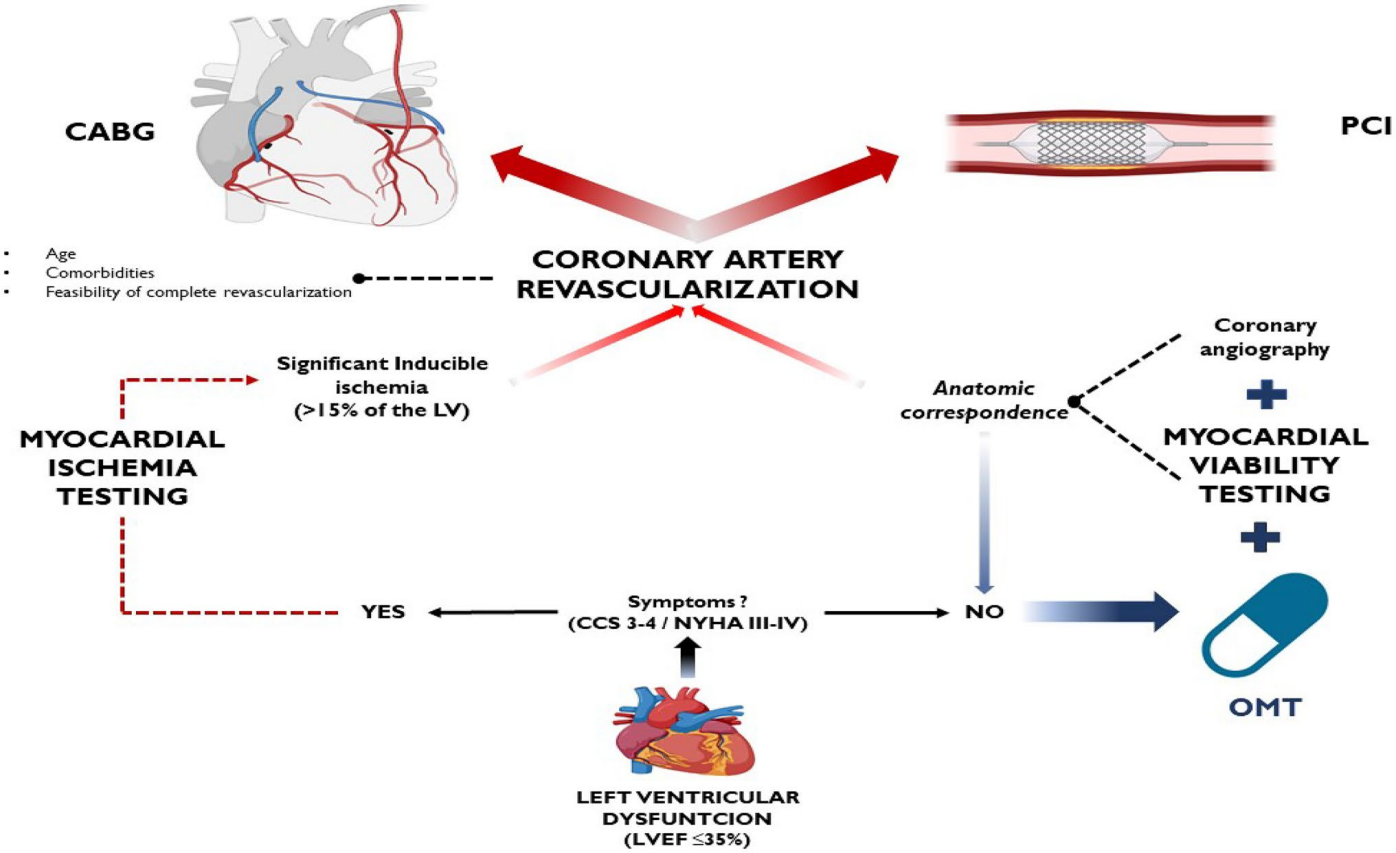
Therapeutic-diagnostic algorithm proposal for left ventricular dysfunction. CABG coronary artery bypass grafting, PCI percutaneous coronary intervention, OMT optimal medical therapy, NYHA New York Heart Association, CCS Canadian Cardiovascular Society angina classes, LV left ventricle. Assessing symptoms should be the first step in the comprehensive evaluation of left ventricular dysfunction: symptomatic patients (NYHA III–IV, CCS 3–4) should perform a myocardial ischemia testing and consider coronary artery revascularization (CABG preferred to PCI) only if there is significant inducible ischemia (i.e., > 15% of the left ventricle). By contrast, asymptomatic or paucisymptomatic patients (NYHA I–II, CCS 1–2) should optimize medical therapy as default strategies and then consider coronary artery revascularization only in case of anatomic correspondence between viable segments and coronaries that are suitable for revascularization: age, comorbidities, and feasibility of complete revascularization should be assessed to evaluate the best reperfusion strategy

The terrific improvements in OMT over the past two decades, along with the introduction of novel medications shown to improve the prognosis for patients with CAD and heart failure (either in isolation or in combination), have called into question the contemporary role of coronary revascularization in this clinical setting. The STICH trial indicates that CABG has a survival benefit over an extended 10-year follow-up period, although this benefit was not significant in the initial 5-year follow-up period. More recently, the findings of the REVIVED-BCIS2 trial indicate that PCI provides no survival benefit over OMT; however, extended follow-up and additional secondary analyses are warranted to identify patient subgroups that might benefit from PCI. In contemporary practice, patients with ischemic cardiomyopathy still experience a high mortality rate, and this trend underscores the urgent need to identify the best therapeutic approach for each patient (i.e., CABG, PCI, or OMT alone). The available data suggest that comorbidities, the age of the patient, the relationship between myocardial viable segments and coronary arteries suitable for revascularization, and the feasibility of complete revascularization may be key considerations for selecting and guiding the optimal treatment strategy (Fig. 1). Given the uncertainty surrounding the available evidence, further investigations are needed to better understand the role of coronary revascularization in patients with ischemic cardiomyopathy.

## Data Availability

All data and materials used are available online.
